# Mental health stigma among nursing students: current status and intervention strategies— an integrative review

**DOI:** 10.3389/fpsyt.2026.1831406

**Published:** 2026-07-06

**Authors:** Weiwei Wang, Xinhang Chen, Mingzhu Liu, Peiwei Zou, Junhua Ye, Ahmad Naqib Shuid

**Affiliations:** 1 Institut Perubatan dan Pergigian Termaju, Universiti Sains Malaysia, Penang, Malaysia; 2 Nanchang Institute of Technology, Nanchang, Jiangxi, China; 3 Fuzhou Medical College of Nanchang University, Fuzhou, Jiangxi, China

**Keywords:** intervention, mental health, nursing student, review, stigma

## Abstract

**Objective:**

Aims to synthesize the current evidence on the prevalence of mental health stigma among nursing students and to summarize the interventions that have been developed and evaluated to reduce such stigma.

**Methods:**

A comprehensive search was conducted across PubMed, CINAHL, and Web of Science databases for peer-reviewed studies published in English between January 2018 and June 2024.

**Results:**

A total of 25 studies were included. The prevalence of mental health stigma among nursing students was found to be moderate to high. Stigma manifested in various forms, including negative attitudes toward patients, internalized shame, and public stigma directed at nursing students themselves. Interventions such as mental health training, empathy enhancement programs, and clinical internships demonstrated varying degrees of effectiveness in reducing stigma, with multi-component interventions showing the most promise.

**Conclusion:**

Mental health stigma among nursing students is a persistent and multifaceted issue that requires targeted strategies to address. Enhancing mental health education, increasing clinical exposure, and implementing comprehensive intervention programs are essential steps to reduce stigma and support the development of compassionate, skilled nursing professionals.

## 1 Introduction

According to a report by the World Mental Health Organization, approximately one in two people will experience a mental health by the age of 75 (1). Recent data from the World Health Organization and other organizations indicate a significant increase in the number of people living with mental health worldwide, with around 100 million individuals affected (2). At the same time, disability and mortality rates are rising, leading to an increased disease burden and economic losses (3).

Mental health are often characterized by unpredictable behavior, a tendency toward violence, long disease courses, difficulty in achieving full recovery, and a high risk of relapse (4). As a result, the public frequently holds stereotypical views toward individuals with mental health, manifesting as prejudice and discrimination—phenomena referred to in the medical field as stigma. The development of stigma can have adverse effects on disease recovery, social values, empowerment, and more (5, 6). An editorial in The Lancet highlighted that mental health impacts areas such as political participation, charitable donations, and local services (7). As members of the general public, nursing students may also hold mental health stigma attitudes toward mental health (8–10).

Nursing students, as the backbone of future healthcare services, hold attitudes and perceptions toward mental health that not only harm the interests of patients (11, 12), but also hinder their own professional development (13). Furthermore, such attitudes perpetuate stigma within the nursing profession, creating a self-fulfilling cycle (14). Existing studies indicate that most nursing students do not pursue mental health work after graduation (15), and mental health stigma is a key reason for the low uptake of mental health specialties (16), remaining a significant societal challenge today (17). Simonelli-Muñoz et al. (18) emphasize that, as future nurses, students must learn, assess, and address skills related to mental health stigma. It is crucial for them to provide care with the utmost neutrality and without discrimination—an aspect often neglected by society. Additionally, nursing students play an essential role in normalizing and destigmatizing mental health, helping to promote acceptance and social integration for individuals with mental health conditions (19, 20).

Mental health stigma remains a fundamental barrier to improving global mental health; however, there is limited understanding regarding how to achieve its sustained reduction (11). Although a previous review (21) has provided a comprehensive overview of this issue, it was published only as a protocol, with the full text not yet available, and its focus differs from the present work. That review primarily analyzed the influencing factors and did not address intervention strategies. Analyzing only the influencing factors offers limited guidance for the development of interventions aimed at reducing stigma. Moreover, as research on interventions continues to increase, there is a need to summarize their scientific validity and feasibility. However, to date, no studies have specifically explored this topic. Accordingly, the present literature review aims to explore the current status of mental health stigma among nursing students and discuss potential interventions.

## 2 Methods

This study utilized an integrative review design to synthesize evidence concerning mental health stigma among nursing students. Preferred Reporting Items for Systematic Reviews and Meta-Analyses (PRISMA) was followed to ensure the reporting quality of this review (22). A review protocol was developed by the research team before the literature search and data extraction were conducted; however, it was not formally registered in a public registry such as PROSPERO or the Open Science Framework. It examined the status of stigma and the existing interventions, incorporating studies with diverse methodologies to offer a comprehensive understanding of the subject (23). A comprehensive search was conducted in PubMed, CINAHL and Web of Science databases. **Table 1** shows the search term categories.

**Table 1:** Search term categories.

Concept	Keywords
Symptom	mental disorder OR mental illness OR mental health OR mental disease
Sample	nurs* OR nurses OR nursing OR nurse
Group	student OR undergraduate OR postgraduate
Management	assessment OR evaluation OR intervention OR management
Performance	stigma OR stereotype OR attitude OR discriminate OR prejudice

### 2.1 Inclusion exclusion criteria

Inclusion criteria were limited to peer-reviewed studies published in English between January 2018 and June 2024, this allowed us to focus in on recent and relevant literature on this topic, focusing on nursing students and mental health stigma using qualitative, quantitative, or mixed-methods approaches. The mental health stigma among nursing students was examined, with particular attention given to the interventions relevant to this population. Articles were excluded from consideration if they were published in languages other than English or if they were unpublished manuscripts, such as abstracts or dissertations. Additionally, a comprehensive search was conducted using Google Scholar. Furthermore, a manual examination of the reference lists from the studies included in the review was performed to identify seminal works within the field. Notably, no restrictions were imposed on the date range for these *post-hoc* searches.

### 2.2 Search outcomes

**Figure 1:**
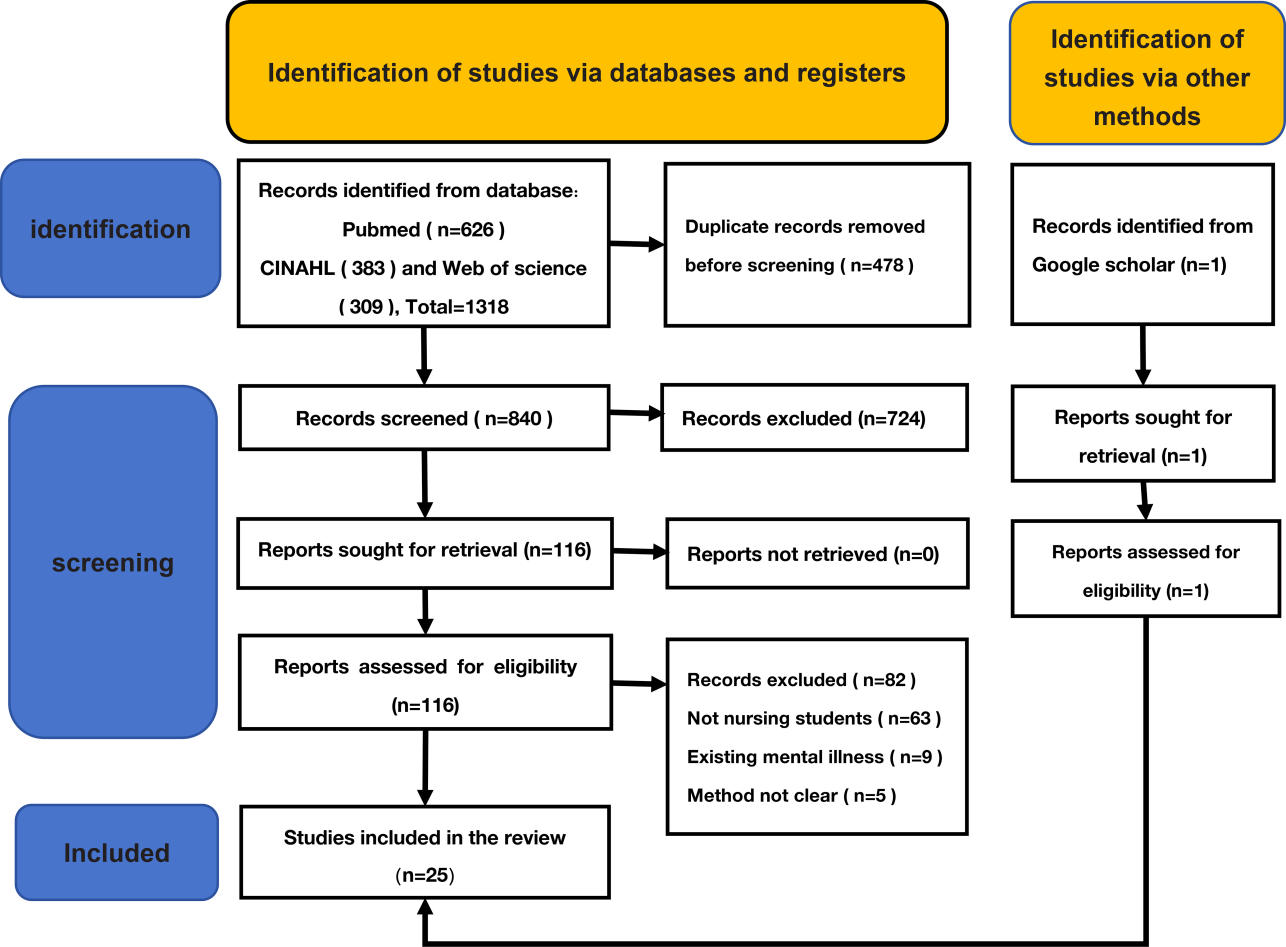
PRISMA flow diagram.

A total of 1318 records were retrieved. After removing duplicates, 840 articles remained. After initial screening by title and abstract, 116 articles remained. Two members of this research team completed independent review and coded the articles as “include”, “exclude” or “maybe”. The independently coded articles were merged into one document to evaluate the reviewer’s decision. If the two reviewers agreed, they followed it. If the reviewers marked differently or marked it as “maybe”, it was decided by group discussion. Finally, 25 articles were included. The PRISMA flow diagram (**Figure 1**) provides an overview of the review stages.

### 2.3 Data evaluation

The study selection process entailed two independent reviewers systematically screening articles based on their titles and abstracts, subsequently proceeding to a full-text evaluation. Duplicate records were eliminated utilizing Zotero. A standardized data extraction form was employed to systematically collect study characteristics and findings. The quality of the included studies was appraised using the Joanna Briggs Institute Critical Appraisal Tools. Ultimately, a thematic analysis approach was adopted to synthesize the extracted data and identify recurring themes and patterns. As the study relied on secondary data derived from published literature, ethical approval was not requisite.

### 2.4 Data analysis and presentation

The data analysis employed the integrative review methodology as outlined by Whittemore and Knafl (23). For the literature that satisfied the inclusion criteria, the researcher conducted a thorough reading, classification, and data extraction process, subsequently organizing the findings into a comprehensive review table. The data were presented using a review table specifically developed by the researcher, which included details such as the first author, publish year, country and setting, aim, methodology, instrument, findings. This structured method ensures a comprehensive and transparent presentation of evidence and helps to gain a deeper understanding of the research on mental health stigma among nursing students. **Table 2** shows the complete articles.

**Table 2:** Characteristics of included studies.

No	First author, year	Country and setting	Aim	Methodology	Instrument	Findings
1	Hawthornes et al., 2020 (24)	Scotland, Practical nurses (adult health, mental health, child health and learning disabilities) and midwives	To explore the level of stigma among students in various fields of nursing and midwifery of different age groups and to examine the impact of exposure to patients with mental illness.	Quantitative, cross-sectional	Community Attitude toward the Mentally Ill (CAMI)	Stigma increases with less contact with people with mental illness, but improves with more contact.
2	Fernandes et al., 2022 (25)	Portugal, Nursing undergraduate	assess the stigma nursing students have towards people with mental illness and examine the relationship between stigma and psycho-socio-demographic variables, so that intervention actions that allow changing this panorama can be developed.	Quantitative, cross-sectional	Attribution Questionnaire (AQ)	Education alone in a classroom setting is not enough to reduce stigma among nursing students, clinical placements in the field are needed to achieve such results. Nursing curricula worldwide must be improved so that students are exposed to psychiatric nursing theory and clinical practice in the first year of their nursing degree.
3	Schoening et al., 2021 (26)	US, Nursing students and nurses	The purpose of this study was to describe the student, staff nurse, and patient experience in a PMH DEU.	Qualitative, focus group interview method	Interview questions	Participation in the PMH DEU helped break down the stigma surrounding mental illness. Nurses described the benefits of staying up-to-date and having a faculty presence. Both student and faculty nurses identified teamwork and understanding PMH nursing roles as key themes.
4	EI Hayek et al., 2021 (27)	Lebanon, Faculty Medical Students Nursing Students	To explore factors associated with mental health stigma in a private university and its affiliated tertiary care center in Lebanon.	Quantitative, cross-sectional	Opening Minds Scale for Health Care Providers (OMS-HC)	There are mixed patterns of discriminatory behaviours and perceptions towards mental illness. This highlights the need for mental health awareness campaigns and interventions in the country.
5	Lim et al., 2020 (28)	Singapore, Nursing students	undergraduate nursing students' perceptions toward mental health clinical placement experiences, degree of clinical confidence and stigma surrounding mental illness.	Quantitative, cross-sectional	Social Distance Scale (SDS)	Attitudes toward clinical internship experiences were significantly associated with stigma attitudes and clinical confidence, with willingness to work in a mental health setting significantly associated with stigma attitudes and attitudes toward clinical internship experiences, emphasizing a stronger sense of preparedness, less anxiety, and a greater preference for a future career in mental health nursing.
6	Simonelli et al., 2023 (18)	Spain, Students in the last year of their nursing degree	The purpose of the present study was to learn about nursing students' perceptions of providing care to patients with severe mental disorders before and after participating in a simulated student clinical case.	Qualitative, semi-structured interviews	Interview questions	The use of high-fidelity simulation provides nursing students with the opportunity to approach patients with mental health conditions, overcome their fears and normalize mental disorders. Simulation training enables nursing students to analyze the reasoning of clinical judgment and discover the impact of previous biases about mental illness on their clinical decision-making.
7	Valentim et al., 2023 (29)	Portugal,Senior Undergraduate Nursing Students	This study explored senior undergraduate nursing students’ perceptions of mental health and its stigma by simulating a case of a patient with mental health issues.	Qualitative, focus group interview method	Interview questions	The findings show that stigma manifests itself in multiple ways, both at the individual and collective levels, suggesting that stigma impedes the well-being of people with mental illness. The individual manifestations of stigma are related to its impact on the person with mental illness, while at the collective level, it is related to the family or society at large.
8	Giralt et al., 2022 (30)	Spain, Nursing undergraduate	To analyze the expected behaviors and attitudes toward MHPs in a group of nursing students undergoing mental health training, taking into account the influence of social desirability.	Quantitative, repeated-measures	Attribution Questionnaire (AQ)	The results showed that the clinical practice stage, due to the proximity to care for people with mental health problems, improves attitudes and behaviours towards mental health in students who have not had mental health problems, and also in younger students.
9	Foster et al., 2019 (31)	Australia and Europe, Nursing undergraduate	This study aimed to investigate undergraduate nurses’ attitudes towards stigma and recovery from mental illness and to describe their understandings of personal recovery as they enter and exit traditional mental health clinical placements.	Quantitative, single-group quasi-experimental study	Opening Minds Scale for Health Care Providers (OMS-HC)	Mental health clinical placements are effective in improving students’ mental health stigma and attitudes towards recovery and provide an excellent opportunity to attract students to the field. Co-production or consumer-led education provided by peer workers during clinical placements may improve students’ stigma attitudes and inspire their interest in pursuing a career in the field.
10	Chiu et al., 2022 (32)	China, Nursing undergraduate	To investigate the effect of name change of schizophrenia on stigma among nursing students. To investigate public stigma, self-stigma and social distance related to schizophrenia and compare the situation before and after the name change.	Quantitative, cross-sectional	Attribution Questionnaire (AQ)	The renaming of schizophrenia has reduced the stigma associated with it. Providing accurate information, guidance from qualified mentors, and access to patients with acute exacerbations in hospital settings and recovering patients in the community are essential.
11	Linz et al., 2022 (33)	US, Nursing undergraduate	The process of recovery was explored through digital stories created by service users, with support from nursing students. Stigmatized attitudes in nursing students and stigma resistance in service users were also investigated.	Mixed research	Opening Minds Scale for Health Care Providers (OMS-HC)	Statistical significance was not found, however, qualitative analysis revealed recovery elements and greater appreciation of individuals with mental illness by the nursing students.
12	Rodriguez et al., 2019 (34)	Spain, Nursing undergraduate	The aim of this study was to determine the extent and associated factors of stigma towards people with mental health problems among students pursuing a nursing degree.	Mixed research	Mental Health Stigma Scale (MHSS)	Students had moderate levels of perceived stigma. Lower levels of stigma were found among students in grades 3 and 4 (i.e., after receiving mental health training) and students who had a family member with a mental health problem.
13	Foster et al., 2021 (31)	Australia, Nursing students	To explore nursing students' experiences of traditional mental health clinical placements and how this placement influences their practice and understanding of recovery from mental illness.	Qualitative, focus group interview method	Interview questions	Through positive placement experiences, students feel supported and included by staff, begin to see patients as people rather than illness diagnoses, gain a deeper understanding of mental health nursing work, and are more likely to consider mental health nursing as a career option. Peer support workers have a significant influence on students' understanding of recovery and play a key role in students' placement education. Students need help and support from university and clinical staff to deal with vicarious trauma that may occur during placements. Mental health placements play a vital role in attracting students to the field, so it is important that they are included in comprehensive pre-registration education.
14	Roach et al., 2023 (35)	US, Students were enrolled in a public academic health science center undergraduate nursing program.	the purpose of this analysis was to use nursing student reflections on their back grounds and perceptions related to mental health stigma to better support students in their learning about mental health topics and inform future pedagogy on mental health in undergraduate nursing education.	Qualitative, focus group interview method	Interview questions	Although improvements have been made to address mental health stigma, stigma still exists. Using student reflections to examine backgrounds and perceptions on mental health stigma provides important insight into student experiences that is helpful in informing pedagogy around mental health curricula and may be a way to mitigate stigma and support students in making sound clinical judgments for people with mental illness.
15	Wedgeworth et al., 2020 (36)	US, Nursing students	To understand undergraduate nursing students’ goals and perceptions of mental health prior to a mental health course.	Qualitative, semi-structured interviews	Interview questions	Nursing students were primarily concerned about their ability to communicate effectively with mental health patients, leading to concerns about upcoming mental health internships. Students also discussed various stigmas surrounding mental health patients and illnesses. Students enter mental health courses and internships with various preconceptions. Nurse educators play a central role in identifying and developing psychoeducational strategies to address student concerns and increase student interest in mental health nursing.
16	Richards et al., 2023 (37)	US, Nursing students	To determine the impact of undergraduate mental health nursing courses on students’ attitudes toward individuals with mental illness, depression, and schizophrenia.	Quantitative, single-group quasi-experimental study	The Prejudice toward People with Mental Illness, Shortened Version (PPMI-SV)	Mental health courses resulted in modest reductions in prejudice. However, some aspects of prejudice remained unchanged. Significant curricular reform is needed to optimize the impact of undergraduate nursing education.
17	Martinez et al., 2019 (38)	Spain, Nursing students	The aim of this study was to measure whether a direct contact intervention, developed in the classroom and involving people with lived experience with mental illnesses as practitioners, produced a change in the emotions and negative attitudes of nursing students towards this group.	Quantitative, single-group quasi-experimental study	Attribution Questionnaire (AQ)	The mental health stigma of nursing students could be improved through direct contact interventions with people diagnosed with mental health disorders, in which an immediate family member also takes part.
18	Moxham et al., 2024 (39)	Transnational,Nursing students	To compare attitudes toward mental illness among undergraduate nursing students in six countries: Australia, India, Jordan, Saudi Arabia, Taiwan, and the United States.	Quantitative, cross-sectional	Social Distance Scale (SDS)	Nursing students’ attitudes toward mental illness differed across countries. Nursing students from Jordan and Saudi Arabia had the highest social distance scores. Students from Taiwan and India had moderate stigma scores. Students from the United States and Australia had the lowest social distance scores. There were clear differences in mental health stigma between countries; these are discussed in relation to possible cultural influences.
19	O'Ferrall et al., 2020 (40)	Spain, Nursing students	To identify factors associated with the evolution of attitudes toward mental health stigma among a group of students undergoing mental health training.	Quantitative, prospective study	Attitude to Mental Illness (AMI)	Academic performance, online consultation, and the size or source of the theory group were indicators of better attitudes. Positive evolution of attitudes was not sustained over time.
20	Graham et al., 2020 (41)	Canada, Nursing students	Research on the role and uniqueness of psychiatric nursing. The main theme of integrated knowledge of mental health, mental illness, and addiction is the basis for applying sub-themes such as therapeutic relationship, holistic approach, recovery orientation, stigma reduction, and advocacy for change.	Qualitative, focus group interview method	Interview questions	As psychiatric nurses continue to move into new areas of health care delivery, students will require preparation and education related to the traditional aspects of psychiatric care including psychopathology, psychotherapeutic interven tions, as well as psychiatric rehabilitation and recovery. More advanced and specialized nursing skills may be taught as required depending on the workplace.
21	Cho and Kim, 2024 (42)	Korea, Nursing students	focused on nursing students and examined the effects of an empathy enhancement program on communication, social distance, and prejudice for people with mental illness.	Quasi-experimental comparative pre–post study	Social Distance Scale (SDS)	Both the EEP-PS group and the clinical placement group showed improvements in communication, social distance, and prejudice toward patients with mental illness. Direct patient interaction and the use of patient narratives as an indirect approach are effective ways to enhance nursing students' attitudes toward mental illness and reduce stigma toward mental illness.
22	Perlman et al., 2019 (43)	Australian, Nursing students	The aim was to examine whether students’ self-determination influenced their level of professional learning during clinical placements, as measured by their stigmatizing attitudes.	Quantitative, single-group quasi-experimental study	Social Distance Scale (SDS)	Students with high levels of self-determination about their work had less stigmatizing attitudes after completing their clinical placement. The results of this study provide empirical evidence that personality elements such as self-determination and work motivation can play a role in the education of future professionals.
23	Sengun et al., 2021 (44)	Turkey, Nursing students	The purpose of this study was to investigate the impact of a peer education program on nursing students’ beliefs about mental illness and their career choices	Quantitative, single-group quasi-experimental study	Beliefs Toward Mental Illness Scale (BMI)	The peer education program had a positive impact on nursing students' beliefs about mental illness. After the peer education program, it was determined that students preferred psychiatric nursing as a career field.
24	Gu et al., 2021 (45)	China, Nursing students	The objective was to examine the effects of psychiatric mental health education through role-playing and real-world encounters on Chinese nursing students’ stigma toward people with mental illness.	Quantitative, analytical cross-sectional mediation study	The Stigma towards People with Mental Illness Scale (SPMI)	Psychiatric mental health education, especially incorporating role-playing and real-world contact, is an effective way to reduce nursing students' stigma and negative attitudes toward patients with mental illness and increase their willingness to care for patients with mental illness.
25	Rodriguez et al., 2022 (46)	Spain, Nursing students	This study aimed to create and apply an educational escape room to train nursing students in mental health and promote positive attitudes toward patients with mental disorders.	Quantitative, quasi-experimental longitudinal study	Attribution Questionnaire (AQ)	The students who participated in the study received better scores in sensitization, and these scores remained better over time. The escape rooms used are suitable for the training and sensitization of future care professionals in the field of mental health, promoting the learning of knowledge and positive attitudes towards serious mental disorders.

## 3 Result

### 3.1 Characteristics of the studies

This review encompasses a total of 25 articles, comprising 16 quantitative studies (7 cross-sectional, 8 quasi-experimental before-and-after studies, and 1 prospective study), 7 qualitative studies, and 2 mixed-method studies. The majority of these studies were conducted in Spain (n=6), the United States (n=5), and China (n=2). Notably, most studies focused exclusively on nursing students, with only 5 studies incorporating participants from other groups, such as nurses and medical students, alongside nursing students. The characteristics of the studies are detailed in **Table 2**.

### 3.2 Current status of mental health stigma

The cross-sectional studies, along with certain qualitative investigations included in this review, generally reported mental health stigma towards mental health among nursing students; however, the extent of such mental health stigma varied considerably across different countries and cultural contexts. The overall findings indicated that nursing students in Jordan and Saudi Arabia exhibited a high level of mental health stigma (39), whereas levels of mental health stigma in China (32, 47), India (34), Singapore (28), and Spain (48) were moderate. In contrast, nursing students in the United States (39) and Australia (9, 34) demonstrated the lowest levels of mental health stigma. These variations may be attributable to the differing degrees of negative stereotypes surrounding mental health within the sociocultural environments of these countries.

### 3.3 Identified intervention approaches​​

Effectively addressing mental health stigma among nursing students, with the aim of reducing or even eliminating such attitudes, is a current research priority. This section will comprehensively examine four key intervention strategies: theoretical education, digital education, peer support, and clinical practice.

#### 3.3.1 Diversification of theoretical education

Educational interventions are a key strategy in reducing mental health stigma among nursing students. However, theoretical guidance alone is often insufficient to deeply challenge students’ stereotypes and emotional attitudes. Research indicates that the impact on mental health stigma may weaken over time (40).

Effective stigma reduction requires the integration of diversified theoretical education with experiential pedagogical approaches. Specific implementation modalities encompass multiple parallel strategies: establishing Dedicated Education Units (DEUs) (26), implementing co-creative educational frameworks wherein mental health survivors participate directly as co-educators (42), conducting structured classroom lectures, facilitating patient narrative sharing sessions, and organizing focused group discussions (41, 46).

Reflective writing is also widely recognized as an effective intervention method (9, 16, 35, 36, 43). This method is suitable for most educational settings.

#### 3.3.2 Digital education

Innovative digital pedagogical approaches afford nursing students immersive, interactive learning experiences that more effectively dismantle stigmatizing barriers. Such methodologies encompass ‘escape room’ teaching strategies (46), autonomous online learning modules (40), and digital storytelling interventions (33). These approaches are particularly suitable for educational institutions with robust technological infrastructure.

#### 3.3.3 Peer support

For nursing students, while guidance from educators remains crucial, peer support represents an equally significant factor that warrants careful consideration. In practice, establishing egalitarian, non-authoritative learning environments—such as organizing peer discussion groups or mutual learning circles—enables students to engage in open and meaningful discourse regarding mental health topics. This approach facilitates their recognition of inherent biases, enhances empathetic understanding, and effectively diminishes negative stereotypes (44), while simultaneously increasing career selection in psychiatric nursing (31).

#### 3.3.4 Clinical practice

The majority of studies in this review examined the impact of contact interventions on stigma reduction (24, 27, 28, 30, 32, 37). Research consistently demonstrates that contact experiences exert significant positive effects on nursing students’ mental health stigma, facilitating transformation of negative perceptions toward mental health (38) and enhancing their willingness to provide psychiatric care (32). Corroborating evidence indicates that nursing students with greater willingness to engage in mental health care typically report lower levels of mental health stigma (25, 45).

However, contact effects may follow a “J-curve” trajectory, whereby initial exposure (32), virtual contact (24), or encounters with severe psychiatric presentations (36) can precipitate vicarious trauma among nursing students, resulting in psychological distress (31) and potentially reinforcing mental health stigma. Regarding long-term sustainability, the absence of continuous support may lead some students to revert to previous attitudes following contact termination. Additionally, prolonged contact exposure carries risks of psychological stress and professional burnout (36). Consequently, Valentim et al. (29) emphasize that integrating contact interventions with educational components yields superior outcomes (30, 37).

## 4 Findings

### 4.1 The ongoing challenge of mental health stigma among nursing students

The findings reaffirm that, despite ongoing efforts to enhance mental health awareness and education (5), mental health stigma among nursing students remains a significant issue, prevalent across various countries and educational contexts. The studies reviewed indicate that levels of stigma among nursing students are moderate to high, with notable regional and cultural variations (39). Nursing students may act as both perpetrators and recipients of stigma, frequently internalizing negative societal attitudes, which can manifest as reluctance to engage in mental health nursing or feelings of shame associated with psychiatric nursing (49). This observation is congruent with Goffman’s conceptualization of stigma as a dynamic, socially constructed process (50), and aligns with the multidimensional frameworks proposed by Rüsch et al. (51) and Link and Phelan (52).

### 4.2 Intervention approaches for reducing mental health stigma

#### 4.2.1 Educational foundations for addressing mental health stigma

Educational interventions are a crucial means of reducing mental health stigma among nursing students. By imparting knowledge, reshaping attitudes, and developing practical skills, such interventions help students to establish a scientific understanding and a professional approach to mental health care. Theoretical courses constitute the core of educational training interventions, providing nursing students with a comprehensive and structured knowledge base regarding mental health. Research indicates that nursing students with higher academic achievement tend to exhibit lower levels of mental health stigma (40). Foundational learning should be deepened through a variety of educational strategies (26, 41, 46). For example, the creation of learning units or the adoption of co-creative educational approaches can not only help to dispel stereotypes, but also enhance teamwork, professional identity, and foster positive interactive experiences.

#### 4.2.2 Advances in educational strategies for addressing stigma

Purely theoretical education has been shown to have limited impact. Innovative educational approaches offer nursing students immersive and interactive learning experiences, stimulating their interest, deepening retention of course content, and cultivating empathy, thereby more effectively breaking down barriers of prejudice. Linz et al. (33) utilized multimedia technology to present “digital characters” sharing patients’ personal experiences, a method which both protects patient privacy and increases the engagement of learning. Simonelli (18) employed high-fidelity simulation to create realistic patient scenarios, providing students with a safe learning environment in which to reduce stigma. Additionally, role-play exercises (36) encourage students to adopt alternative perspectives, fostering empathy by allowing them to better understand the challenges faced by patients, and thus reducing mental health stigma.

These innovative intervention strategies offer a range of options for addressing mental health stigma among nursing students and demonstrate considerable potential to enrich teaching methods and enhance intervention effectiveness. However, the wider implementation of such strategies is constrained by cost and technical complexity. Their scope of application and long-term effectiveness require further investigation, particularly in relation to models that integrate these approaches with traditional interventions.

#### 4.2.3 Bridging theory and practice: addressing stigma through multifaceted educational approaches

The long-term impact of purely theoretical exposure on the mental health stigma is limited (25). According to the knowledge-attitude-behavior model, the combination of theoretical and innovative educational approaches can only exert an initial effect at the cognitive level, while clinical practice is the crucial stage for translating knowledge into practical competence.

In addition to diversifying teaching content, classroom formats are continually evolving, with attention given to factors that influence nursing students’ learning outcomes, such as self-determination, empathy, resilience, and self-care strategies (25, 31, 43). These approaches serve to correct misconceptions about mental health and lay a cognitive foundation for subsequent clinical practice. Reflective writing, which has received increasing attention in recent years, is one such intervention (9, 18, 35, 36, 43). It offers students a safe space in which to articulate their biases and misunderstandings regarding mental health issues. Reflective writing enables students to identify and gradually challenge their own stereotypes, thereby reducing mental health stigma towards individuals with mental health.

It is therefore essential that nursing education incorporates cultural sensitivity training, enabling future nurses to appreciate the concept of mental health within diverse cultural contexts and to provide more inclusive care. The formation of mental health stigma among nursing students is shaped by the combined influence of multiple factors, both independent and interactive, which collectively inform their attitudes towards individuals with mental health. Future research should further investigate the mechanisms underlying these interactions and develop targeted interventions tailored to specific cultural and educational contexts, in order to effectively reduce mental health stigma among nursing students.

#### 4.2.4 Sustaining change: the role of contact, education, and context in reducing mental health stigma

Contact-based interventions are an important strategy for reducing mental health stigma among nursing students. At their core, such interventions involve direct interaction with individuals living with mental illness, which challenges stereotypes and fosters empathy. Contact-based interventions have generally been associated with more positive attitudes on nursing students’ attitudes, with direct contact in particular shown to markedly reduce fear and avoidance behaviors, as well as increase students’ willingness to provide care (32, 38). However, observational studies have also suggested an inverse association between willingness to provide care and the degree of mental health stigma. (25, 45).

It is important to consider the methods, timing, and context of contact-based interventions. Across the included studies, immediate improvements were reported more frequently than sustained long-term effects, partly because many studies assessed stigma only before and immediately after the intervention. Brief or isolated contact may therefore produce short-term improvements, but these effects may weaken over time without ongoing support, structured reflection, and curricular reinforcement. For example, O'Ferrall-González et al. (40) reported that positive changes in attitudes were not sustained over time, whereas Rodríguez-Ferrer et al. (46) found that improvements after a digital escape-room intervention were partly maintained at follow-up. Some studies also indicated possible unintended challenges, such as increased anxiety or stigma during initial contact with patients in acute states (32). Therefore, combining contact with educational components, guided reflection, and supportive supervision may produce more consistent improvements (29, 30, 37).

Overall, multicomponent interventions appeared to be the most promising approach, particularly when theoretical education was combined with direct contact, clinical placement, simulation, guided reflection, peer involvement, or experiential digital learning. This interpretation is supported by the pattern of findings showing that interventions addressing cognitive, emotional, and behavioral dimensions of stigma tended to produce more consistent improvements than single-component approaches. However, this conclusion should be interpreted cautiously because the included studies varied in design, stigma instruments, outcome domains, and follow-up duration. Reducing stigma is therefore important not only for improving students’ attitudes, but also for supporting professional identity formation, career choice, therapeutic relationships, and the quality of future mental health care.

### 4.3 Limitations and strengths

Despite these advances, several limitations remain. Many studies rely on self-reported measures of stigma, which may be influenced by social desirability bias. This review also has certain limitations. The majority of included studies are cross-sectional, limiting causal inference. The exclusion of non-English publications may have resulted in language bias. Additionally, variations in the definition and measurement of stigma across studies may contribute to heterogeneity in the findings. This integrative review possesses several notable strengths. The included articles are highly relevant to the research objectives, and an integrative review approach was adopted to systematically examine the current state and interventions related to mental health stigma among nursing students. The review encompasses a wide range of study types and international literature, ensuring comprehensive coverage and methodological rigor.

## 5 Conclusions and clinical implications

This review found that mental health stigma is a prevalent and multifaceted issue among nursing students, with most studies reporting moderate to high levels of stigma. Intervention studies targeting mental health stigma among nursing students have demonstrated the effectiveness of various strategies, including contact, education and training, and peer education. Importantly, these studies highlight that the core of stigma reduction lies in achieving comprehensive change across cognitive, emotional, and behavioral domains. It is noteworthy that the effectiveness of such interventions is closely linked to a profound understanding of cultural context and educational environment.

Future research and practice should focus on constructing an integrated intervention framework that spans the entire nursing education process. This framework should organically combine contact, education, and peer support, establishing a structured training system aimed at cultivating nursing professionals equipped with both expertise and humanistic care. Ultimately, such an approach seeks to fundamentally eliminate mental health stigma and bring about long-term positive change in the field of mental health nursing.

The findings of this review have clear implications for nursing education and policy. Integrating anti-stigma content into undergraduate curricula, providing meaningful clinical experience in mental health settings, and promoting open dialogue about mental health are key steps towards reducing stigma. Active guidance from educators and institutional support are equally vital for fostering an inclusive learning environment. Future research should focus on the rigorous evaluation of intervention effectiveness, including long-term follow-up and the exploration of specific contextual factors.

## Data Availability

The original contributions presented in the study are included in the article/supplementary material. Further inquiries can be directed to the corresponding author/s.
